# Listeria monocytogenes Brain Abscess Presenting With Stroke-Like Symptoms: A Case Report

**DOI:** 10.7759/cureus.52216

**Published:** 2024-01-13

**Authors:** Roxana M Dragomir, Olivia Mattner, Veronica Hagan, Marc A Swerdloff

**Affiliations:** 1 Neurology, Florida Atlantic University Charles E. Schmidt College of Medicine, Boca Raton, USA; 2 Internal Medicine, Florida Atlantic University Charles E. Schmidt College of Medicine, Boca Raton, USA

**Keywords:** treatment, diagnosis, case report, listeria, brain abscess

## Abstract

We present a case of *Listeria monocytogenes *brain abscess in an immunocompromised patient admitted for stroke-like symptoms of headache and aphasia. Computerized tomography of the head revealed a 1.7 x 1.3 cm left frontal lobe lesion with surrounding edema, secondary to stroke, tumor, or abscess. Magnetic resonance imaging brain revealed a ring-enhancing lesion and a small contralateral area of restricted diffusion. Two of the two blood cultures grew an organism identified as *L. monocytogenes *using matrix-assisted laser desorption ionization time-of-flight mass spectrometry. Treatment with ampicillin and trimethoprim-sulfa yielded marked symptomatic improvement. A brain biopsy was consistent with bacterial abscess. The patient’s clinical course was favorable, with improved aphasia and negative follow-up blood cultures. A literature review found a limited number of *L. monocytogenes *abscess cases and none had clear guidelines for diagnosis. Recent studies have proposed five criteria for diagnosis. Our patient fulfilled three of these proposed guidelines.

## Introduction

Patients presenting with stroke-like symptoms must undergo an urgent stroke protocol imaging to maximize the chances for thrombolytic therapy or mechanical thrombectomy. When a stroke has been eliminated as a possibility, then a more thorough medical and travel history (especially in immunocompromised males in their fifth to sixth decade) should be undertaken. Blood cultures are an important link in the diagnosis. *Listeria monocytogenes* is notoriously difficult to isolate using standard blood culture techniques. Matrix-assisted laser desorption ionization time-of-flight mass spectrometry (MALDI-TOF MS) is an important advance in identifying this fastidious organism from blood cultures, by comparing unique microorganism mass spectral fingerprints with a large library of mass spectra for accurate microbial identification.

*L. monocytogenes* is a gram-positive facultatively anaerobe bacteria found in soil, water, or raw products. It is a cryophilic organism transmitted via contaminated food, particularly afflicting pregnant women, the immunocompromised, and those at the extremes of age [1,2]. Immunocompetent individuals often experience self-limiting gastroenteritis, while the immunocompromised may have high mortality from sepsis, meningitis, and rhombencephalitis [3]. The organism invades the central nervous system (CNS), most frequently the medulla oblongata and pons, and infrequently, the supratentorial white matter and cerebellar hemispheres [4].

About 10% of CNS infections with *L. monocytogenes *are complicated by the development of abscesses with mortality three times higher than other organisms [5-7]. It is hypothesized that the abscess forms by infiltrating the CNS via capillary endothelial cells or infectious macrophages penetrating the blood-brain barrier [6].

## Case presentation

A 64-year-old dextral male presented to our hospital with two episodes, each lasting under one minute, of the sharp right parietal headache of 5/10 intensity followed by transient aphasia. Stroke risk factors were age, hypertension, hyperlipidemia, and stage III chronic kidney disease. He was on chronic immunosuppression for Lupus nephritis with mycophenolate and prednisone. He denied prior similar symptoms, weakness, paresthesia, fever, or chills. Two weeks before, he had self-limited traveler’s diarrhea while on a Caribbean vacation.

He was afebrile, normotensive, alert, and oriented to person, place, time, and situation. He had aphasia, with impaired repetition, skipping occasional letters, intermittent word-finding difficulty with the retained ability to name common objects and their subparts. He had normal language comprehension. There was no dysarthria or anosognosia. He was able to follow complex commands that crossed the midline. The rest of the neurological examination was unremarkable, including no frontal lobe signs of abulia, apathy, or change in personality.

He had 86% granulocytes, a white cell count of 9.3 K/mcL, an erythrocyte sedimentation rate of 22 mm/hr, glomerular filtration rate of 38 mL/min/1.73 m^2^, microscopic hematuria, negative rapid human immunosuppression virus test (Table 1).

**Table 1:** Laboratory findings LDL: Low-density lipoprotein

Laboratory	value	unit	Normal range
Granulocytes	86	%	42-75
White cell count	9.3	K/mcL	4-10
Erythrocyte sedimentation rate	22	mm/hr	0-25
Glomerular filtration rate	38	mL/min/1.73 m^2^	>60
LDL	66	mg/dL	<129
HbA1c	5.8	%	3.8-5.6

**Figure 1:**
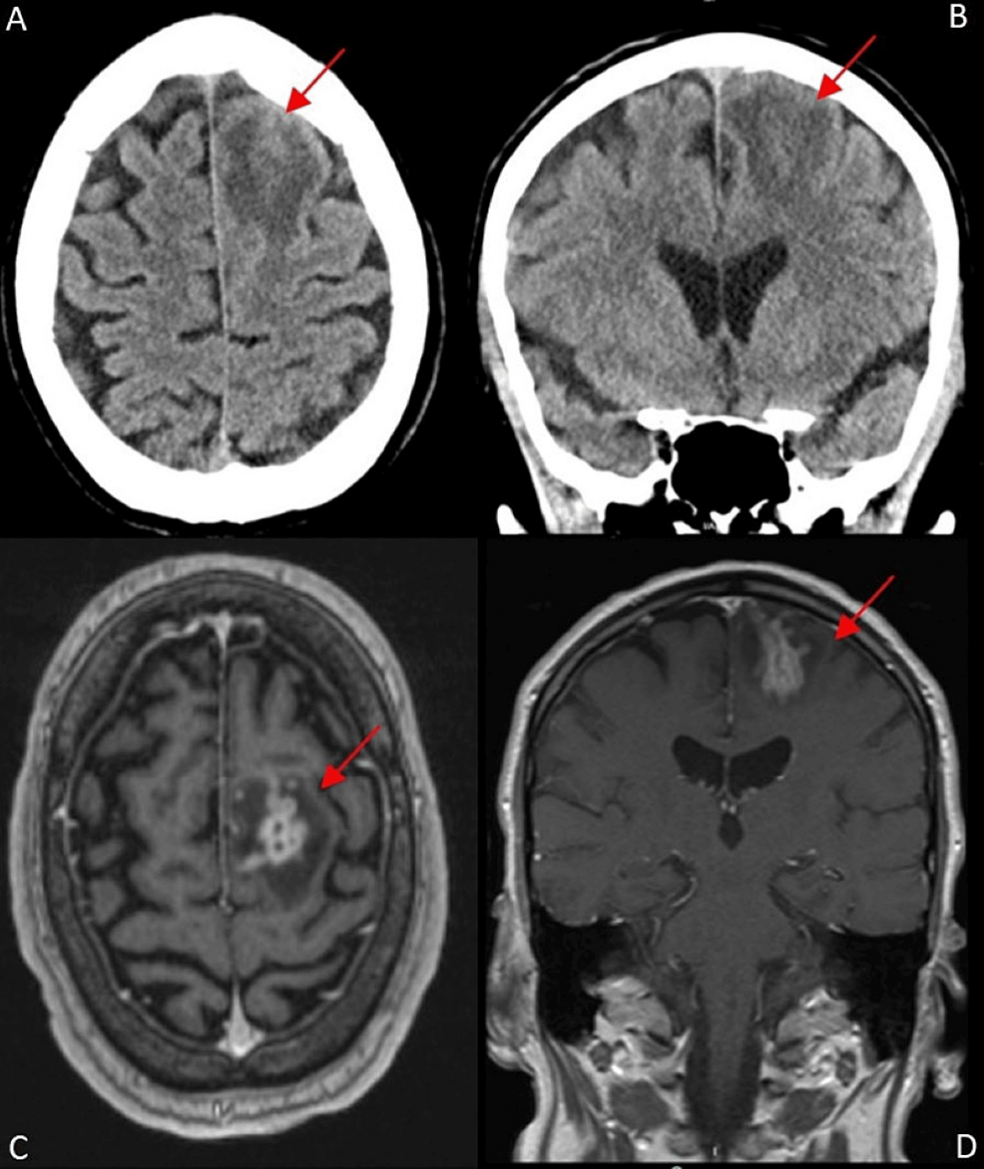
Axial and coronal CT head and MRI revealing left intra-axial frontal lobe lesion with edema Panel A: axial CT head without contrast, panel B: coronal CT head without contrast, revealing a left intra-axial frontal lobe lesion measuring 1.7 x 1.3 cm with surrounding edema (arrow), panel C: axial MRI brain with contrast, and panel D: coronal cuts revealing an intra-axial peripherally enhancing lesion centered in the medial posterior left frontal lobe surrounded by vasogenic edema with some areas of tubular appearance measuring 1.6 x 1.9 x 2.2 cm (arrow) with minimal mass effect and no midline shift.

A plain CT scan of the head (Figure 1) revealed a left intra-axial frontal lobe lesion measuring 1.7 x 1.3 cm with surrounding edema, minimal mass effect, and no midline shift. CT scans of the abdomen, chest, and pelvis were negative for systemic malignancy.

**Figure 2:**
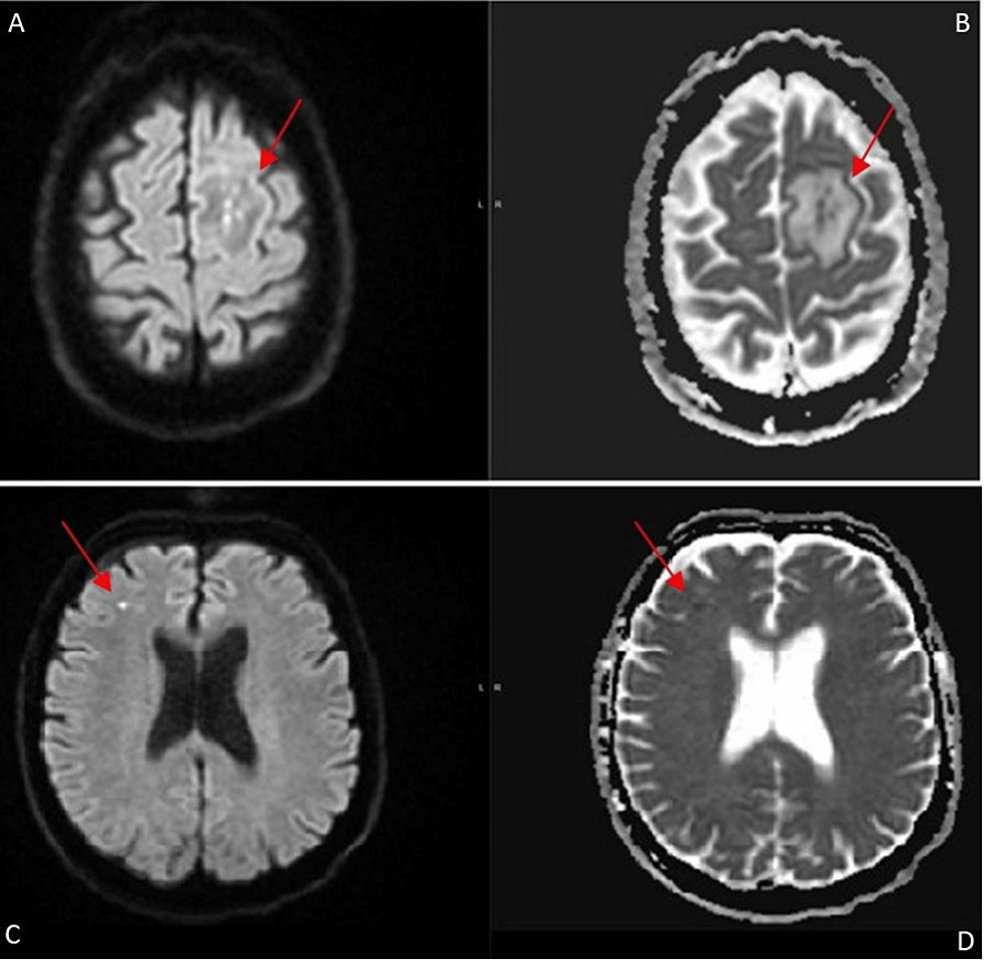
MRI brain findings MRI brain with contrast, axial cuts. Panel A: diffusion-weighted imaging (DWI), panel B: apparent diffusion coefficient (ADC) revealing scattered areas of diffusion restriction (arrow) in the center of the left frontal lesion, panel C: additional small punctiform area of diffusion restriction in the right frontal lobe (arrow), and panel D: corresponding ADC (arrow) concerning acute infarcts from an embolic source. Not presented, however there was no evidence of blood products on the susceptibility images.

An MRI of the brain with contrast (Figure 1) showed a peripherally enhancing left frontal intra-axial mass, in the medial posterior left frontal lobe, surrounded by vasogenic edema suspicious for neoplasm vs. abscess. There was a second punctiform area of diffusion restriction in the right frontal lobe consistent with an acute embolic infarct (Figure 2).

CT angiography showed normal arteries, including intra and extracranial carotids, vertebrobasilar, anterior and middle cerebral, and posterior communicators. Electroencephalogram showed intermittent delta slowing over the left hemisphere consistent with underlying subcortical abnormality. Blood culture analysis using MALDI-TOF MS had two of two blood cultures identified as *L. monocytogenes*. Treatment with ampicillin and trimethoprim-sulfa was initiated resulting in marked symptomatic improvement.

**Figure 3:**
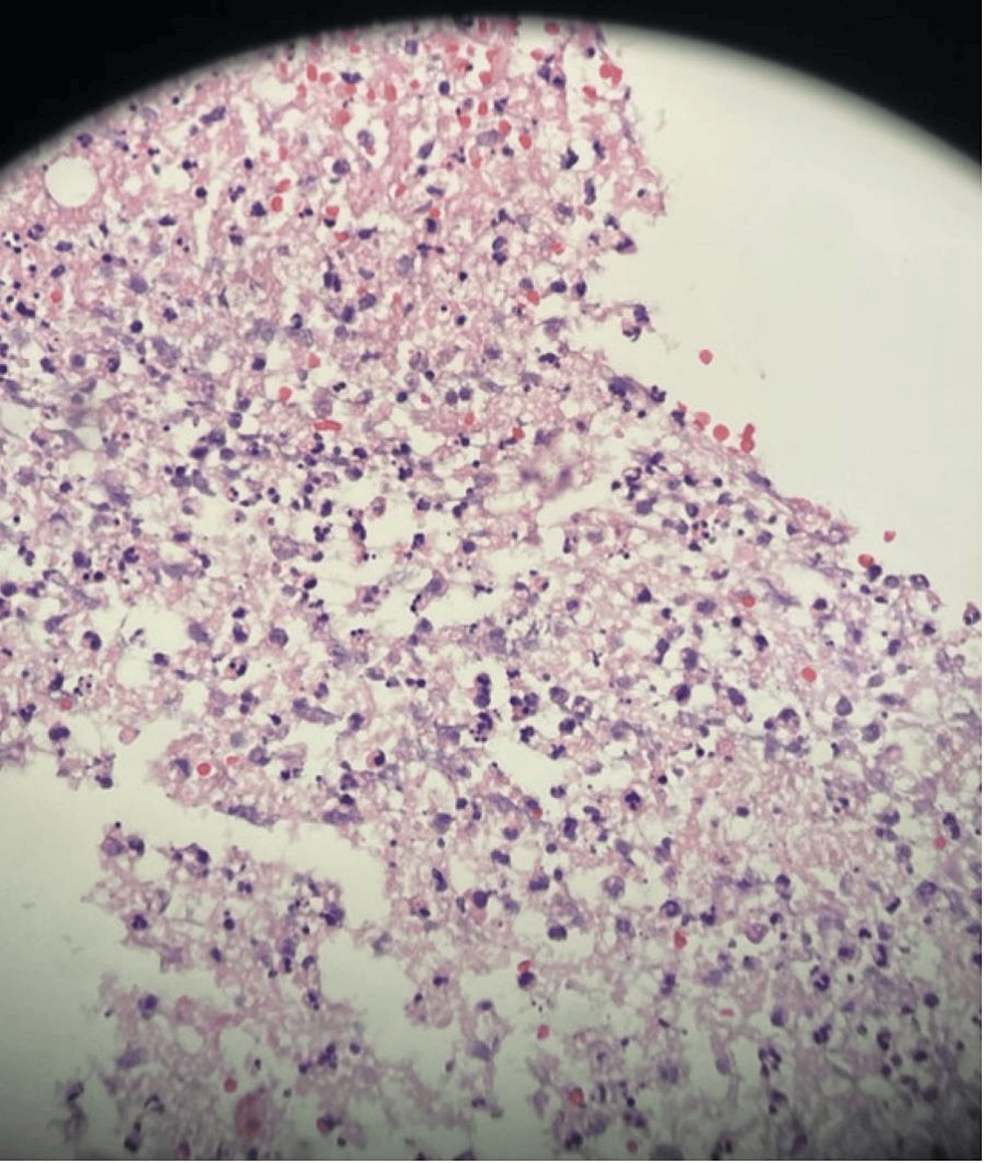
Surgical pathology histologic examination Surgical pathology - magnification 100 oil: histologic examination reveals abscess associated with reactive gliosis, microhemorrhage, and very focal perivascular lymphocytosis. No morphologic evidence of glioma, metastatic carcinoma, lymphoma, granulomatous inflammation, or viral cytopathic effect.

His neurosurgeon added dexamethasone and for seizure prophylaxis, levetiracetam, prior to a biopsy of the lesion. He underwent a left frontal craniotomy with partial resection of the lesion using intraoperative neuro-navigation and intraoperative electrophysiological monitoring for motor cortex mapping. The gross pathology sample was described as whitish, soft, and friable, without overt purulence. The microscopic pathology showed an abscess associated with reactive gliosis, microhemorrhage, and focal perivascular lymphocytosis (Figure 3).

The patient’s clinical course was favorable, with improvement in aphasia and resolution of headache. Follow-up blood cultures were negative.

He was discharged home on ampicillin, trimethoprim-sulfa, levetiracetam, dexamethasone, and mycophenolate. He was directed to follow up with neurosurgery, neurology, and infectious disease clinics.

## Discussion

Intracranial abscesses may develop insidiously. The clinical manifestations of* L. monocytogenes *brain abscesses are nonspecific and are due to acute infection. The patients may present with classic signs like fever, headache, increased intracranial pressure, or focal signs, mimicking stroke or tumor. Depending on the location of the abscess there may be word-finding difficulty and paresis [6,8-11]. Importantly, these patients may be asymptomatic, without classic infectious signs (granulocytosis or fever). This may cause a delay in diagnosis and therapy, increasing morbidity and mortality.

The diagnosis of brain abscess has significantly improved over the past decades with advances in neuroimaging allowing early detection and characterization. A contrast CT head can show the size and location of the abscess with a surrounding ring of enhancement [12,13]. MRI, using diffusion weighting sequences, increases sensitivity and specificity to over 90%, distinguishing infection from neoplasm. An abscess containing viscous cellular pus will show restricted diffusion and low apparent diffusion coefficient, in contrast to neoplasm, though not all abscesses follow this rule [14]. *L. monocytogenes* abscesses are nearly always associated with bacteremia and 25% of cases have *L. monocytogenes* isolated in the CSF [5].

Recent studies have proposed five points to guide diagnosis: acute onset, impaired cell-mediated immunity, or changes in eating habits with or without preceding infection; fever as a first symptom with or without headache, nausea, meningeal signs, focal neurological dysfunction; MRI imaging consistent with abscess; exclusion of other types of mass or other bacterial abscess; and positive blood or CSF cultures [15].

Affected patients are in their fifth to sixth decade of life, 61.9% were male, and 47% were treated with ampicillin monotherapy [15]. The most common therapy was high-dose ampicillin with gentamicin, followed by trimethoprim/sulfamethoxazole and vancomycin [7]. In some studies, patients with surgical drainage using stereotactic aspiration or craniotomy had a higher survival rate compared to antibiotic treatment alone. Other studies recommended using a surgical approach based on abscess size (greater than 2.5 cm) and location, depending on the judgment of the treating surgeon [14,15].

## Conclusions

The overall incidence of brain abscesses is estimated at 1-2% in Western countries and 8% in developing countries. There are 87 cases of *L. monocytogenes* brain abscess reported between 1968 and 2021. The majority were male patients, with one or more risk factors, more likely treated with multiple antibiotics than monotherapy, with or without surgery. Several cases presented with initial stroke-like symptoms of speech difficulty and paresis. Corticosteroid treatment was a predisposing factor for *L. monocytogenes* abscess. Diagnosis required an elevated level of suspicion with a multidisciplinary approach between neurology, infectious disease, and neurosurgery. As in the literature, our patient was a male in his sixth decade of life, with corticosteroid immunosuppression, who presented with aphasia and headache. A comprehensive history, physical, and laboratory workup was of paramount importance coupled with advanced neuroimaging and microbiology. MALDI-TOF MS advanced analysis more rapidly and accurately isolated *L. monocytogenes* from blood cultures than in the past. Recent studies have proposed five criteria for diagnosis. Our patient fulfilled three of these proposed guidelines. In the proper clinical setting, one should have a high index of suspicion for *L. monocytogenes* infection as these criteria need to be further validated before actually being used.
